# Dietary Supplementation with Silicon-Enriched Spirulina Improves Arterial Remodeling and Function in Hypertensive Rats

**DOI:** 10.3390/nu11112574

**Published:** 2019-10-25

**Authors:** Joanna Arthur-Ataam, Patrice Bideaux, Azzouz Charrabi, Pierre Sicard, Bérengère Fromy, Kiaoling Liu, Saadia Eddahibi, Côme Pasqualin, Nicolas Jouy, Sylvain Richard, Anne Virsolvy

**Affiliations:** 1 PhyMedExp, Université de Montpellier, INSERM U1046, CNRS UMR9214, 34295 Montpellier, France; joanna.arthur@hotmail.fr (J.A.-A.); patrice.bideaux@inserm.fr (P.B.); azzouz.charrabi@inserm.fr (A.C.); pierre.sicard@inserm.fr (P.S.); saadia.eddahibi@inserm.fr (S.E.); sylvain.richard@inserm.fr (S.R.); 2 CNRS UMR 5305, Université Claude Bernard Lyon 1, 69367 Lyon, France; berengere.fromy@univ-lyon1.fr (B.F.); kiaoling.liu-lo@univ-lyon1.fr (K.L.); 3 EA 7349 Université de Tours, 37200 Tours, France; come.pasqualin@univ-tours.fr; 4 Phyco-Biotech, 34070 Montpellier, France; nicolas.jouy@umontpellier.fr

**Keywords:** spirulina, silicon, hypertension, arterial structure, vascular function

## Abstract

Vascular aging is characterized by increase in arterial stiffness and remodeling of the arterial wall with a loss of elastic properties. Silicon is an essential trace element highly present in arteries. It is involved in the constitution and stabilization of elastin fibers. The nutritional supply and bioavailability of silicon are often inadequate. Spirulina (Sp), micro algae have recognized nutritional properties and are able to incorporate minerals in a bioavailable form. We evaluated the effects of nutritional supplementation with silicon-enriched spirulina (SpSi) on arterial system structure and function in hypertension. Experiments were performed on hypertensive (SHR) and normotensive Wistar-Kyoto (WKY) rats supplemented with SpSi or Sp over a period of three months. Arterial pressure, vascular function and morphometric parameters of thoracic aorta were analyzed. SpSi supplementation lowered arterial pressure in SHR and minimized morphometric alterations induced by hypertension. Aortic wall thickness and elastic fibers fragmentation were partially reversed. Collagen and elastin levels were increased in association with extracellular matrix degradation decrease. Vascular reactivity was improved with better contractile and vasorelaxant responses to various agonists. No changes were observed in SHR supplemented with Sp. The beneficial effects of SpSi supplementation evidenced here, may be attributable to Si enrichment and offer interesting opportunities to prevent cardiovascular risks.

## 1. Introduction

Silicon (Si), a major trace element and essential nutrient for humans, is beneficial in bone formation and connective tissue health especially vascular tissue [1,2]. Aorta, tendons, skin, and bones are rich in Si with aortic tissue containing four to five times more Si than liver, heart, and muscle tissues in rats [3]. Si content significantly declines with age in aorta and connective tissues in several animal species [4] and humans [5,6], especially women [7]. This reported decrease with age and disease progression could be related to a potential deficiency in intake. Silicon was established as an essential nutrient since the 1970’s, and compelling evidence continues to accumulate indicating that silicon contributes to health [8]. Data from human and animal studies demonstrate that dietary silicon increases bone mineralization and collagen synthesis, improves structural integrity of connective tissue and reduces the risk of atherosclerosis [9,10,11,12]. Food is the major source of silicon for humans with higher intakes obtained from plant-based foods rather than animal products [13]. However, due to changes in dietary practices, silicon intake in humans has decreased [14]. Supplementation with bioavailable forms of Si has proven therapeutic potential in the prevention of degenerative processes, especially bone demineralization and atherosclerosis [15]. Moreover, besides intake level, the bioavailability of Si is of paramount importance and not always established. Si exists in mineral and soluble forms. The mineral form (SiO_2_) is insoluble and poorly available. The water soluble form which is absorbed by humans is the orthosilicic acid (Si(OH)_4_). It is obtained from fluids (water, beer) or by hydrolysis of silicon from solid foods in the gastrointestinal tract. Food is the major source of available Si and much of the silicon consumed is poorly soluble with limited nutritional impact. Thus, the development of new functional nutrients to improve nutritional status and mitigate nutritional deficiencies is of interest.

New types of food supplements based on organic biomass such as yeast, lactobacilli, or spirulina, have been developed and could serve as rich sources of bioavailable trace elements [16]. Among them, Spirulina (Sp) is emerging as a potential resource of functional food. Sp is a species of filamentous cyanobacteria that has long been consumed as a food supplement. The genius, Spirulina platensis, is primarily used due to its high protein and vitamin contents [17]. Apart from its nutritional and health properties, Sp possesses the ability to incorporate and accumulate trace elements in a soluble form that is bioavailable in mammals. This has been previously shown for selenium, iron and manganese [16]. Incorporating the soluble form of Si into spirulina is a way to obtain a bioavailable food supplement providing the beneficial effects of silicon and the antioxidant and anti-inflammatory properties of Sp [18]. The beneficial effects of silicon enriched-spirulina (SpSi) were previously demonstrated on disorders and dysfunctions induced by a high-fat diet in the Syrian hamster model [19]. The positive effects on vascular function were especially attributed to Si enrichment. It is well established that obesity is associated with functional and structural changes in the vascular wall [20]. Such structural changes occur during hypertension, resulting in increased arterial stiffness and decreased vascular compliance. The reduction of elastic properties is associated with the remodeling and rearrangement of structural main components of the vascular wall and extracellular matrix [21]. The most predominant mechanisms thought to underlie these changes are an increase in collagen deposition and a breakdown of elastin [22].

Considering the biological role of Si, we evaluated the effects of SpSi nutritional supplementation on cardiovascular system alterations induced by hypertension in the spontaneously hypertensive rat (SHR). 

## 2. Materials and Methods 

### 2.1. Spirulina and Tablet Supplements

Spirulina platensis (Sp) and silicon-enriched Spirulina platensis (SpSi) were produced by PhycoBiotech (Montpellier, France). The complete production process of SpSi is protected by a patent filed by Phyco-Biotech. Sp were grown in raceway ponds in Zarouk’s medium at 22 °C and pH 10.5 and in the presence of 1 g/L sodium metasilicate (Na_2_SiO_3_) for Si-enrichment as described [19]. The biomass recovered by filtration was then washed, cut, quickly dried at 42 °C in a vented dryer, finely grinded and used as a powder. As indicated by the manufacturer, the final Si contents were 2% for Sp-Si and less than 0.023% (Si detection limit) for Sp and orthosilic acid Si(OH)_4_ is the predominant form present in spirulina.

Spirulina powders (Sp and SpSi) were used to prepare the dietary supplements as previously described [23]. Compact dough made by mixing Sp or SpSi powders with standard rat chow (Safe Diet A03, France) and water, was spread and cut into 3 mm diameter discs. The tablets obtained after drying at 40 °C for 24 hours had a constant weight of 30 ± 2 mg. Their Sp or SpSi content was determined relatively to the Si daily need estimated around 40 mg for humans (70 kg) with a correspondence to 15 mg Sp and 0.3 mg Si (2%) for a 500 g rat. Tablets composition was adapted to the body weight of the rat throughout the experiment (400–500 g). Control tablets were prepared exclusively with standard rat chow (0.2% Si).

### 2.2. Animals and Experimental Design

Experiments were performed on 12 weeks old male normotensive Wistar-Kyoto (*n* = 15) and hypertensive SHR (*n* = 45) rats housed under conditions of controlled temperature (22–24 °C), humidity (45%–50%) and light cycle (12 light hours/12 dark hours) with standard food and tap water ad libitum. Hypertensive animals were randomly separated in three groups of 15 individuals supplemented for 12 weeks with either control (SHR) or Sp (SHR-Sp) or SpSi (SHR-SpSi) tablets. Normotensive animals were supplemented with control tablets (WKY). Tablets were administered daily by gavage with small forceps. Body weight was monitored weekly. All experimental procedures were conducted in accordance with the European Union Laboratory Animal Care Rules (2010/63/EU Directive) and were approved by the local ethical committee of Montpellier (APAFIS#3922-2016020317052023).

### 2.3. Arterial Pressure

At the end of the protocol, animals were anesthetized by intraperitoneal injection of pentobarbital (Invasive blood pressure was measured via cannulation of the right carotid artery using a pressure transducer connected to a monitor (AD Instruments, Paris, France). Diastolic and systolic arterial pressures were recorded.

### 2.4. Sampling and Plasma Assay

Animals were sacrificed by exsanguination through the pressure catheter. The thoracic aorta was removed immersed in PSS buffer and cleaned of fat and connective tissue. Aortic tissue was then cut into 2–3 mm width rings used either directly for vascular reactivity experiments, fixed in 4% PFA for histological analysis or frozen in liquid nitrogen then sored at −80 °C for further RNA extraction. Blood samples were centrifuged for 20 minutes at 4000 g at 4 °C and plasma were frozen for further determination of IL-6 and IL-1b with Elisa kit according to manufacturer instructions (Thermo Scientific, Saint Herblain, France).

### 2.5. Echocardiography

High resolution echocardiography (VisualSonics/Fujifilm, Canada with a MS250D ultrasound probe 20 MHz) was performed under anesthesia by 2% isofluorane inhalation at 37 °C, and under ECG and respiratory rate monitoring, as previously described [24]. Left ventricular (LV) parasternal long axis 2D views in B-mode and M-Mode were performed at the level of papillary muscle to assess LV wall thicknesses and internal diameters allowing the calculation of the ejection fraction (EF) and LV wall thickness. EF was calculated using Teichholz formula [100 × (LVIDd^3^ − LVIDs^3^)/LVIDd^3^]. The LV mass (corrected) was calculated as LV Mass = 1053 × [(LVIDd+LVPWd+IVSd)^3^ − LVIDd^3^]. Offline image analyses were performed using dedicated VisualSonics VevoLab 3.1.0 software.

### 2.6. Histological Staining and Morphometric Analysis

Thoracic aortic segments of 3 mm length were taken 0.5 cm downstream of the aortic cross, fixed in 4% PFA embedded in paraffin, and cut into 5 µm serial cross-sections with a microtome. Adjacent sections were stained with Verhoeff’s solution, the first step in Van Gieson staining which highlights elastic laminae, or picrosirius red dye (PSR) to examine collagen. Stains were also used for morphometric measurements within the media layer. Optical bright field images were acquired with a slide scanner (Nanozoomer, Hamamatsu) and were digitally analyzed with simple image software (ImageJ, NIH USA) for morphometric analysis and elastin fibers level quantification. Internal and external perimeters of the tunica intima-media were measured using ImageJ to further calculate internal and external diameters and media thickness. Elastin content was determined within each ring after fiber identification by direct pixel quantification using the software. Data are presented as relative density expressed as percentage of the total media section area. Elastin fiber fragmentation was performed with a custom routine running with ImageJ software based on the plugin AnalyseSkeleton [25]. Elastin fibers were detected and fiber fragments were counted and normalized to the analyzed surface area in each preparation.

The PSR-stained sections were imaged under polarized light at × 20 magnification, and birefringent collagen fibers were quantitated (in yellow/red collagen I and in green collagen III). Total collagen contents were determined by direct pixel quantification with ImageJ in a sample field (four fields per section) and normalized with the total media area within the field.

### 2.7. Quantitative PCR

Aortic tissue was homogenized in TRI Reagent (Sigma Aldrich, Saint-Quentin Fallavier, France). RNA was isolated after extraction with chloroform, precipitation with isopropanol and washes with ethanol. RNA was then reverse transcribed with the Verso cDNA Synthesis Kit (Thermo Fisher Scientific, Illkirch, France). Quantitative PCR of cDNA was performed in triplicate with a SYBR Green based assay (Roche Diagnostics, Mannheim, Germany). The following primers were used: collagen1, (F) 5′-AACCTGGATGCCATCAAGG-3′ and (R) 5′- AACTGGAATCCATCGGTCAT-3′; elastin, (F) 5′-GCAGCCTGGCGTCTTG-3′ and (R) 5′-GGATAATAGACTCCACCGGGA-3′; MMP-9, (F) 5′- CTGCAGTGCCCTTGAACTAA-3′ and (R) 5′- TATCCGGCAAACTAGCTCCT-3′. The expression was normalized to endogenous RPLPO (Ribosomal Large Protein) expression (ThermoFischer Scientific, Illkirch, France). The data are reported as relative quantification using the ddCt method. Primer sequences are available as Supporting Information.

### 2.8. Vascular Reactivity

Experiments were performed on freshly isolated rat aortas and as previously described [19]. Arterial segments were mounted between two stainless steel hooks placed in a conventional vertical organ bath chamber filled with 5 mL of PSS, maintained at 37 °C and continuously bubbled with O2. Changes in isometric tension were measured using an IT1-25 force transducer and an IOX computerized system (EMKA Technologies, Paris, France). Each arterial segment was subjected to a 60-min equilibration period at a basal resting tension of 2 g. Arterial contractility was evaluated with phenylephrine (PE, 10^−6^ M). The functionality of the endothelium was assessed by the ability of acetylcholine (Ach, 10^−6^ M) to induce relaxation in PE-contracted rings. After washout and a 20–30 min period of stabilization, the contractile and vasorelaxant abilities of the aorta were respectively evaluated with cumulative increases in the concentration of the depolarizing agent KCl (1 to 80 mM) and PE (10^−9^ to 10^−5^ M) before and after preincubation with N(ω)-nitro-L-arginine methyl ester (L-NAME, 10^−5^ M) or the muscarinic agonists Ach (10^−8^ to 10^−5^ M) and the NO donor sodium nitroprusside (SNP, 10^−9^ to 10^−6^ M). Aortic rings were previously contracted with a submaximal dose of PE (10^−6^ M) prior to Ach and SNP. The rate of relaxation was expressed as the percentage of the PE-induced contraction. 

### 2.9. Statistical Analysis

Four groups were analyzed: normotensive rats supplemented with a control tablet (WKY), hypertensive rats supplemented with a control tablet (SHR), hypertensive rats supplemented with spirulina (SHR-Sp) and hypertensive rats supplemented with silicon-enriched spirulina (SHR-SpSi), *n* = 15 for all groups. Data were expressed as mean ± s.e.m and analyzed using GraphPad Prism (V6.05, RRID:SCR_002798) with one-way ANOVA followed by Tukey’s post-hoc test to compare all groups. Dose-response curves were fitted with nonlinear regressions and statistical differences were assessed using two-way ANOVA followed by Bonferroni’s post-hoc test to compare all groups. Statistical analysis shown compared WKY to SHR treated or not and SHR-SpSi to other groups to evaluate the effects of SpSi. P values lower than 0.05 were considered significant.

## 3. Results

### 3.1. Effect of Supplementation on Blood Pressure

**Figure 1:**
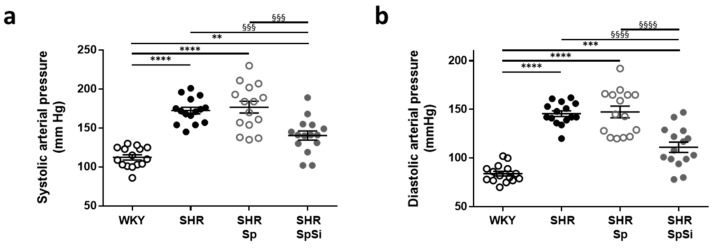
Effect of supplementation on arterial pressure. Blood systolic (**a**) and diastolic (**b**) arterial pressures were measured for all animals in each group (*n* = 15). Values are in mmHg and data are presented as scatter plots with mean ± s.e.m. **** *p* < 0.0001, *** *p* < 0.001, ** *p* < 0.01 vs. WKY; §§§§ *p* < 0.0001, §§§ *p* < 0.001 vs. SHR-SpSi.

Elevated systolic, diastolic and mean arterial pressures validated hypertension in SHR (Figure 1 and Table 1). Systolic and diastolic arterial pressures were 64 mmHg (64%) and 63 mmHg (75%) higher than in WKY, respectively. Animals supplemented with SpSi had mean arterial pressures lower than SHR but still higher than WKY (Table 1). In SHR-SpSi, systolic pressure was 32 mmHg (18%) lower than in SHR and 28 mmHg (25%) higher than in WKY. For diastolic pressure, values were 35 mmHg (24%) lower and 27 mmHg (24%) higher than in SHR and WKY, respectively. No difference was noticed on arterial pressure in the SHR-Sp group vs. SHR. Thus, no effect of Sp supplementation was observed.

### 3.2. Morphometric and Ultrastructural Analysis of the Vascular Wall

**Figure 2:**
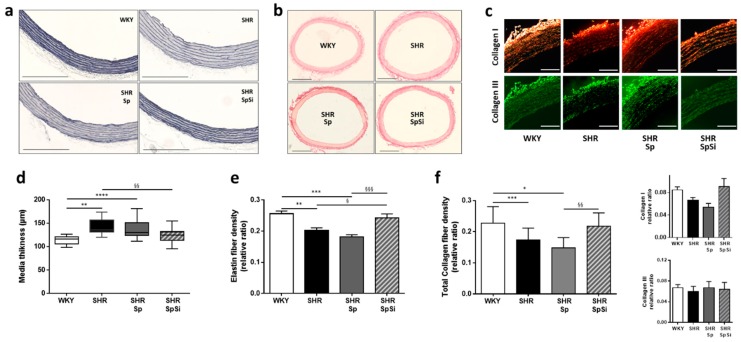
Effect of supplementation on morphology and ultrastructure of aorta. Images show representative histological staining of aortic samples. Bright field images illustrate (**a**) aorta stained with Verhoeff’s solution and evidencing elastin fibers structure and media thickness (scale bar 300 µm); (**b**) aorta stained with picrosirius red and showing internal and external diameters (scale bar 500 µm); (**c**) Picrosirius red stained sections illuminated under polarized light to visualize type I collagen in yellow/red and type III collagen in green (scale bar 100 µm). (**d**) Graph showing media thickness in all groups; data are presented as box and whiskers with median and SD. Bar graphs (**e**) and (**f**) summarize, respectively, elastin and collagen density quantified from 3 to 5 sections per aorta and expressed as relative ratio normalized to media layer surface. Data are presented as mean ± s.e.m (n = 15 in each group). **** *p* < 0.0001, *** *p* < 0.001, ** *p* < 0.01, * *p* < 0.05 vs. WKY; §§§ *p* < 0.001, §§ *p* < 0.01, § *p* < 0.05 vs. SHR-SpSi.

We evaluated morphology of the arterial wall and extracellular matrix structure and composition by histological staining of aortic sections. Elastin staining showed that aortic wall ultrastructure is modified in SHR. We observed increased elastin fiber fragmentation and linearization (Figure 2a). These alterations, still present in SHR-Sp, were normalized in SHR-SpSi. Elastin fragmentation ratio was higher in SHR and SHR-Sp (1.265 ± 0.065 and 1.384 ± 0.098 respectively) than in WKY and SHR-SpSi (0.902 ± 0.062 and 0.853 ± 0.088 respectively) with no difference observed between these two groups (*p* = 0.978), showing efficiency of SpSi supplementation. Moreover, in SHR-SpSi like in WKY, elastin fibers presented multiple refolding and wave shapes that were not observed in SHR and SHR-Sp.

Furthermore, morphometric measurements revealed important changes in the vessel wall of SHR. Media thickness and internal lumen diameter were increased respectively by 25% and 16% when compared to WKY (Figure 2a–d). Internal lumen diameter, measured from 1512 ± 17 µm in WKY to 1758 ± 21 µm (*p* < 0.0001) in SHR. With Sp supplementation no changes were observed for both thickness (Figure 2d) and internal diameter (1719 ± 22 µm, *p* = 0.635) vs. SHR. However, media thickening and aortic wall hypertrophy were both reduced in the SHR group supplemented with SpSi when compared to SHR. Thickening was decreased by 70% and diameter enlarged by 55% (1622 ± 11 µm, *p* < 0.0001). Though, when compare to WKY, those parameters were not completely normalized. Internal diameter remained large in SHR-SpSi group (*p* = 0.0033), yet media hypertrophy was reversed as no difference was observed with WKY for thickness (*p* = 0.386).

### 3.3. Analysis of Extracellular Matrix Components 

**Figure 3:**
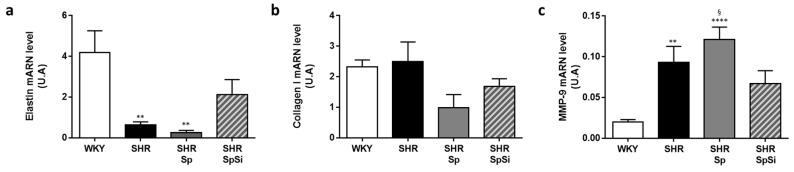
Effect of supplementation on aortic mRNA levels of various extracellular matrix components. The mRNA expression levels were quantified by qPCR in rat aorta for elastin (**a**), collagen I (**b**) and MMP-9 (**c**). Graphs represent mean ± s.e.m of three different determinations in each animal of all groups (*n* = 15). **** *p* < 0.0001, ** *p* < 0.01 vs. WKY; § *p* < 0.05 vs. SpSi.

Si is said to be associated with the elastin and collagen components of the aortic wall matrix. As we observed ultrastructural changes in elastic fibers, we determined elastin and collagen density ratios in our histological stained sections and quantified their mRNA tissue levels. Both elastin and collagen density were decreased in SHR vs. WKY (Figure 2e,f). This was associated with a decrease in mRNA level for elastin (Figure 3a), while no change was found for collagen mRNA level (Figure 3b). SpSi supplementation in SHR corrected elastin and collagen density with ratios that were higher than in SHR (Figure 2e,f). No difference was observed when compared to WKY. For elastin, this was associated to a change in mRNA elastin level which was intermediate between WKY and SHR (Figure 3a). No difference was noticed for collagen mRNA level. Furthermore, no effect of Sp supplementation was found in SHR on both collagen and elastin fiber density and mRNA level.

Matrix metalloproteases (MMPs) participate in vascular tissue remodeling. MMPs activity contributes to the disassembly of intercellular junctions and the degradation of extracellular matrix components. Among them, MMP-9 may be overexpressed in SHR [26]. Thus we also quantified MMP-9 mRNA levels in the different groups. We observed that this level was indeed higher in SHR than in WKY (Figure 3c, *p* = 0.0051). SpSi supplementation reduced MMP-9 mRNA overexpression compared to SHR without reaching significance (*p* = 0.6038). SpSi mRNA level was different from Sp level (*p* = 0.0399) and intermediate between WKY (*p* = 0.0895) and SHR. No difference was observed for SHR-Sp vs. SHR. 

The remodeling of extracellular matrix in SHR consisting of increased degradation and decreased expression of elastin and collagen was partially corrected by SpSi supplementation.

### 3.4. Evaluation of Vascular Contractile Properties of Aorta 

**Figure 4:**
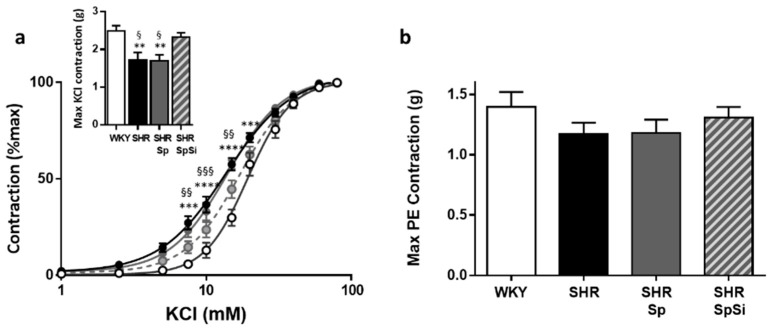
Effect of supplementation on contractile response of aorta. (**a**) Curves summarize cumulative dose-responses to KCl in aortic rings from WKY (white symbol), SHR (black symbol), SHR-Sp (grey symbol) and SHR-SpSi (dotted grey symbol). The inset shows the maximal KCl-induced contraction expressed in grams. (**b**) Bar graph presents the maximal contractions induced by PE in all experimental groups. Data represent mean ± s.e.m (*n* = 15 in each group). **** *p* < 0.0001, *** *p* < 0.001, ** *p* < 0.01 vs. WKY; §§§ *p* < 0.001, §§ *p* < 0.01, § *p* < 0.05 vs. SHR-SpSi.

The contractility of aortic rings was evaluated using the depolarizing agent KCl and the α-adrenergic agonist PE. The contractile response to KCl was impaired in SHR when compared to WKY (Figure 4a). Maximal contractility was decreased by 30 ± 8% in SHR, with lower E_max_ value (*p* = 0.005) (Figure 4a inset and Table 2). In hypertensive animals, we also observed a significant rightward shift in the dose-response curve (Figure 4a) with a diminution of EC_50_ value reflecting an increased sensitivity to KCl-induced depolarization (*p* = 0.0055 vs. WKY, Table 2). These alterations were attenuated by SpSi supplementation. The maximal contractile response to KCl was restored. E_max_ value was higher than that of SHR (*p* = 0.0292) and not different from that of WKY (*p* = 0.8612). KCl-sensitivity was normalized as shown by EC_50_ value being no different from that of WKY (*p* = 0.935) but increased when compared to SHR (*p* = 0.0221). No difference was observed for KCl response and sensitivity between SHR-Sp and SHR with identical E_max_ and EC_50_ values (Table 2). 

However, sensitivity and contractile responses to PE were not changed in SHR when compared to WKY (Figure 4b). E_max_ and EC_50_ values were identical between these groups (Table 2). Therefore, no difference was noticed with Sp and SpSi supplementations. 

**Figure 5:**
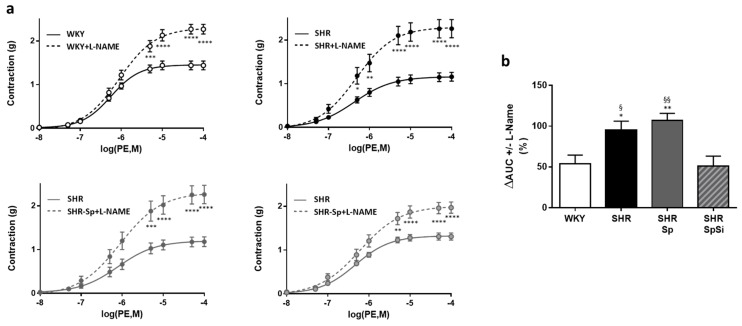
Impact of L-NAME on PE-induced contraction. (**a**) Cumulative dose-response curves to PE were performed in absence (plain lines) and in presence of 10 µM L-NAME (dotted lines). Data are expressed as mean ± s.e.m (*n* = 15). **** *p* < 0.0001, *** *p* < 0.001, ** *p* < 0.01, * *p* < 0.05, PE vs. L-NAME PE. (**b**) Bar graph illustrates the ability of NO to attenuate PE-induced vasoconstriction and corresponded to ΔAUC. It was calculated as the difference in the areas under the concentration response curves of PE in the presence and in the absence of L-NAME and was expressed as percentage of the corresponding AUC for PE. Data are mean ± s.e.m (*n* = 15). ** *p* < 0.01, * *p* < 0.05 vs. WKY; §§ *p* < 0.01, § *p* < 0.05 vs. SHR-SpSi.

To estimate basal NO release in aortic segments, dose-response curves to PE were generated in the presence of L-NAME (Figure 5a). The difference in the contractile level to PE before and after addition of L-NAME would indicate the extent of endothelial basal NO release. In all groups, we observed that L-NAME significantly increased the contractile response to PE (Figure 5a). No difference was observed in E_max_ values. However, the increase was significantly greater in SHR and SHR-Sp than in WKY and SHR-SpSi. This is indicated by the analysis of ΔAUC, defined as the difference in the areas under the concentration response curves of PE in the presence and in the absence of L-NAME. Thus, ΔAUC was higher in SHR and in SHR-Sp than in WKY and in SHR-SpSi (Figure 5b). No difference was found between WKY and SHR-SPSi.

### 3.5. Evaluation of Vasorelaxant Capacity of the Aorta 

**Figure 6:**
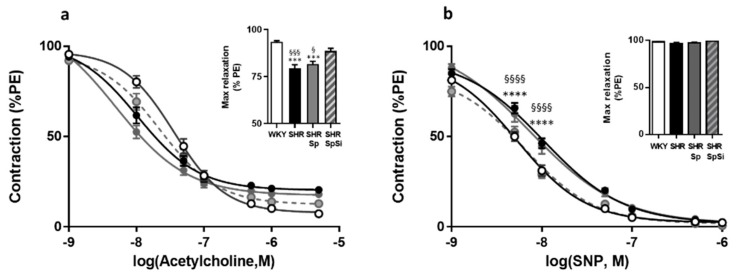
Effect of supplementation on vasorelaxant response of aorta. Cumulative dose response curves to Ach (**a**) and SNP (**b**) in aortic rings in WKY (white symbol), SHR (black symbol), SHR-Sp (grey symbol) and SHR-SpSi (dotted grey symbol). Bar graphs compare maximal relaxations for each antagonist in all groups. Data is expressed as percentage of PE-induced contraction show mean ± s.e.m (*n* = 15). **** *p* < 0.0001, *** *p* < 0.001, vs. WKY; §§§§ *p* < 0.0001, §§§ *p <* 0.001, § *p* < 0.05 vs. SHR-SpSi.

The vasorelaxant properties of the aorta were evaluated with Ach and SNP (Figure 6). Ach assessed functional integrity of the endothelium. With SNP we evaluated the sensitivity of aortic muscle to NO. Ach induced a dose-dependent relaxation that was impaired in SHR (Figure 6a). Both the sensitivity, as assessed by EC_50_ value, and E_max_ of Ach were decreased in SHR when compared to WKY (Table 2). The SpSi supplementation partially restored Ach sensitivity as indicated by increased Emax and EC_50_ values when compared to SHR. They were decreased when compared with WKY, as well. Additionally, we observed that Sp supplementation did not modify the changes evidenced in SHR.

Similarly, SNP induced concentration-dependent relaxation in all groups (Figure 6b). The sensitivity of aortic smooth muscle to the NO donor was decreased in SHR as indicated by lower EC_50_ value (*p* = 0.0009), when E_max_ values were identical (Table 2). SpSi supplementation corrected SNP sensitivity as we observed a lower EC_50_ value in SHR-SpSi than in SHR (*p* = 0.0162) and no difference with WKY (*p* = 0.7784). With Sp supplementation SNP sensitivity was not changed when compared to SHR. 

### 3.6. Analysis of IL-6 and IL-1B Inflammation Markers

**Figure 7:**
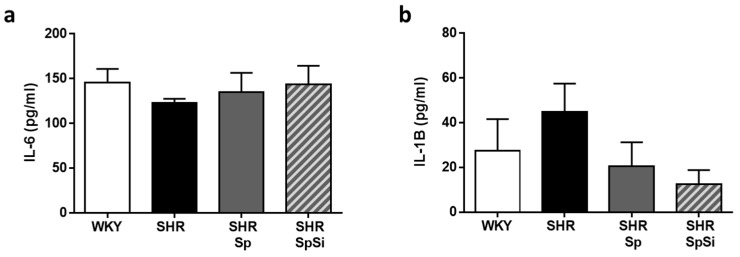
Plasmatic levels of pro-inflammatory cytokines. IL-6 (**a**) and IL-1*β* (**b**) were quantified in plasma. Data are expressed as mean ± s.e.m (*n* = 15).

A link exists between inflammation and hypertension as hypertension is considered a chronic inflammatory disease. Considering the anti-inflammatory properties of Sp [18], we investigated the effects of supplementation on some markers shown to contribute to the pathogenesis of vascular disease (IL-6 and IL-1*β*) [27]. Thus, we quantified mRNA levels in our aortic samples. We found no significant difference in the IL-6 and IL-1*β* expression levels in the aorta of SHR when compared to WKY (Figure 7). No significant variation of mRNA levels was noticed in any group indicating no effect of supplementation.

### 3.7. Effect of Supplementation on Cardiac Morphology and Function

Echocardiography analysis showed increased left ventricular mass and post-wall thickness in SHR (Table 1) indicating hypertrophy of the myocardium. Cardiac function was also altered as evidenced by a decreased ejection fraction (EF). In SHR, EF of 60.3 ± 1.6% was reduced by 13% (*p* = 0.0032) compared to WKY EF of 68.8 ± 2.2%. In animals supplemented with either SpSi or Sp, no change was noticed in those parameters when compared to SHR. In SHR-Spsi and in SHR-Sp, cardiac hypertrophy and dysfunction were not modified.

## 4. Discussion

Considering the properties and biological role of silicon, we evaluated the effects of nutritional supplementation with silicon-enriched spirulina on vascular remodeling induced by hypertension in a SHR rat model. Our study showed highly beneficial effects including attenuation of arterial pressure and improvement of vascular reactivity. These benefits were associated with reduction in arterial thickness and stiffness caused by the structural remodeling of the aortic wall in SHR. They were mainly attributable to Si enrichment.

Beneficial and safe effects of SpSi supplementation were previously reported in high fat diet-induced models of atherosclerosis in hamsters [19] and metabolic syndrome in rats [23]. Improvement of glucose tolerance, insulin resistance, or dyslipidemia were evidenced with both SpSi and Sp supplementation. However, only SpSi had beneficial effects on arterial function [19]. No toxicity or side effects of SpSi supplement were otherwise observed [28]. Increase of arterial stiffness, reduction of arterial compliance, and distensibility marked by fragmentation of elastin are the main consequences of arterial remodeling occurring in hypertension, obesity and aging [29]. Vascular remodeling in hypertension is characterized by altered ultrastructure due to reduction in elastin and increase in fibrous tissue [30]. Here, we assessed the effect of SpSi on the ultrastructural, mechanical and functional changes characterized in SHR thoracic aorta. These modifications consisted of (i) arterial wall hypertrophy, (ii) fragmentation, linearization, and refolding of elastic fibers, and (iii) impaired contractile and vasorelaxant responses. These changes typified the degenerative processes leading to the reduction of compliance and distensibility described in hypertension [22,31,32] and associated with aging [9,33]. Indeed, in SHR aorta, our results showed enlarged internal diameter and media thickening, increased elastin fragmentation ratio, and lower elastin and collagen levels than in normotensive animals. Consequently, contractility was impaired as evidenced by the decreased response and sensitivities to KCl-induced depolarization and PE. The vasorelaxant responses to both Ach and SNP were also reduced. The enhanced effect of L-NAME on PE-induced contraction suggested that the decrease in the vasodilator capacity of aorta in SHR is due to both the alteration of vascular smooth muscle function and NO bioavailability. 

After three months of supplementation, SpSi substantially corrected and even normalized most of the defects identified in SHR aorta. First, aortic hypertrophy was reduced as shown by morphologic analysis. Higher level of elastin fiber density and mRNA level were associated to a decrease in MMP-9 expression, suggesting that both enhanced elastin biosynthesis and reduced extracellular matrix contributed to media thinning. It is known that hypertensive vascular remodeling involves reorganization and impairment of the extracellular matrix and that MMPs play an important role in this remodeling [34]. Second, we observed an improvement of vascular function after SpSi supplementation. Contractile and vasorelaxant responses were increased in association with an increased sensitivity to the agonists and antagonists tested and a better NO bioavailability. Finally, we found that SHR animals supplemented with SpSi had lower arterial pressure than control SHR. Arterial pressure, although significantly reduced, was still higher than normal pressure which may explain the lack of benefit on cardiac parameters in our experimental conditions (hypertension already established, 3 months of supplementation). 

Another interesting result in our study is that supplementation with Sp alone had no effect in SHR suggesting that the benefits observed with SpSi are genuine effects of Si. The nutritional essentiality of Si is undeniable [10,33]. This organic element appears as an important structural component of the aortic wall which contributes to elastin and collagen synthesis and stability [9]. Data from animal and human studies demonstrated that dietary Si mitigates the risk of cardiovascular diseases. Si in drinking water has been inversely correlated with mortality rates due to coronary heart diseases in Finland [11]. Reduced damage and thickening of elastic fibers were observed in the aortic wall after silicon supplementation in rabbit models of diet-induced atherosclerosis [35,36]. Moreover, our results are in line with a previous study reporting reduction in systolic arterial pressure after supplementation with soluble silicon in SHR [37]. In that study, arterial structure was not investigated and enhancement of intracellular magnesium uptake was evoked as a possible mechanism. This is not inconsistent with our findings as Si is supposed to contribute to biological function and affect retention or function of other minerals, such as aluminum or magnesium [8]. Various mechanisms are potentially involved in the beneficial effects of Si. Here, a major result in our study is that SpSi supplementation reverses elastic fiber degradation which is likely to contribute to the overall benefit. The observed regenerative changes of the vascular wall are in line with a Si-related beneficial effect. However, quantification of Si level in the aortic tissue is warranted to validate this hypothesis.

Increase in collagen level is also a result of SpSi supplementation. Collagen increase is often associated with pathological remodeling of arterial structure in hypertension as well as in ageing [38], though this change is not consistently associated with hypertension. Several reports actually show decreases in vascular collagen with hypertension [39,40]. In our study, we found a higher aortic collagen level in SHR-SpSi when compared to SHR but not higher than in WKY. Collagen was not found to be overexpressed when compared to the non-pathological condition, and this was not supposed to be deleterious. Although, we might wonder what the effects of a longer supplementation could be.

In conclusion, the reduction of arterial pressure and the structural and functional changes observed in the aortic walls of SHR rats after SpSi supplementation demonstrated the beneficial effects of this nutritional supplement, especially Si enrichment. The altered vascular phenotype associated with hypertension in SHR was not completely rescued but SpSi nutritional supplementation did ameliorate some of the deleterious effects. In humans, vascular disease and ageing are associated with increased arterial pressure and structural remodeling of arteries which are seen as decline of elasticity and distensibility, fragmentation of elastin and higher collagen-to-elastin ratio. Si supplementation is beneficial in preventing extracellular matrix degenerative processes. Our results show clear beneficial effects of Si (enriched in spirulina) oral supplementation on those degenerative processes. Our results open interesting perspectives for the use of SpSi supplementation in the prevention of vascular degenerative processes and potentially ageing, as well as in different conditions of Si deficiency.

## Figures and Tables

**Table 1:** Effect of supplementation on body weight, arterial pressure and cardiac morphological and functional parameters.

	WKY	SHR	SHR-Sp	SHR-SpSi
Body weight (g)	473.8 ± 6.8	441.1 ± 6.2 **^, §^	464.6 ± 5.8	475.1 ± 10.3
Body weight gain (g)	137.2 ± 4.6	104.6 ± 6.2 *	121.3 ± 8.4	113.2 ± 5.6
Mean arterial pressure (mmHg)	95.2 ± 2.5	157.1 ± 3.2 ****^, §§§§^	160.2 ±6.6 ****^, §§§§^	123.2 ±5.5 ***
Cardiac Frequency (bpm)	348 ± 7	329 ± 7	347 ± 5	339 ± 7
Ejection Fraction (%)	68.8 ± 2.2	60.3 ± 1.6 **	60.8 ± 1.2 **	62.1 ±1.5 *
Left ventricular mass (mg)	851 ± 45	1010 ± 43 *	1041 ± 39	972 ± 43
Post wall thickness, diastole (mm)	1.65 ± 0.06	2.03 ± 0.07 ***	2.19 ± 0.04 ***	2.02 ± 0.06 ***
Post wall thickness, systole (mm)	2.84 ± 0.11	3.17 ± 0.12	3.17 ± 0.07	3.13 ± 0.08

Data are mean ± s.e.m (*n* = 15). **** *p* < 0.0001, *** *p* < 0.001, ** *p* < 0.01, * *p* < 0.05 vs. WKY; ^§§§§^ *p* < 0.0001, ^§^ *p* < 0.05 vs. SHR-SpSi.

**Table 2:** Maximal responses (E_max_) and sensitivity (EC_50_) for the various agonists and antagonists of the contractile response on rat aorta.

	KCl		PE		L-NAME/PE		Ach		SNP	
	E_max_ (g)	EC_50_ (mM)	E_max_ (g)	EC_50_ (nM)	E_max_ (g)	EC_50_ (nM)	E_max_ (%PE)	EC_50_ (µM)	E_10-8_(%PE)	EC_50_ (nM)
WHY	2.49 ± 0.14	19.9 ± 1.4	1.4 ± 0.12	605 ± 81	2.27 ± 0.11	1790 ± 420	93.5 ± 0.6	36.1 ± 5.7	98.6 ± 0.3	5.7 ± 0.7
SHR	1.73 ± 0.19 **^, §^	14.1 ± 0.6 **^, §^	1.17 ± 0.1	550 ± 114	2.26 ± 0.21	1736 ± 677	79.5 ± 1.7 ****^, §§§§^	10.4 ± 2.1	97.1 ± 0.9	10.8 ± 1.5 ***^, §^
SHR-Sp	1.69 ± 0.16 **^, §^	13.9 ± 0.9 **^, §^	1.18 ± 0.11	566 ± 89	2.26 ± 0.21	1827 ± 784	81.6 ± 1.5 ****^,§^	5.7 ± 2.5	97.3 ± 0.9	12.5 ± 0.3 ****^, §§§^
SHR-SpSi	2.33 ± 0.11	18.4 ± 1.04	1.31 ± 0.09	532 ± 62	1.97 ± 0.13	1340 ± 336	88.6 ± 1.4	22.1 ± 4.5	99.3 ± 0.3	6.9 ± 0.6

Data are mean ± s.e.m (*n* = 15). **** *p* < 0.0001, *** *p* < 0.001, ** *p* < 0.01, * *p* < 0.05 vs. WKY; ^§§§§^ *p* < 0.0001, ^§^ *p* < 0.05 vs. SHR-SpSi.
